# Comparative study of individualized 3D-printing navigation technology and free-hand isthmus method in lumbar CBT screw implantation

**DOI:** 10.3389/fsurg.2025.1520481

**Published:** 2025-03-27

**Authors:** Hanlin Hu, Zhenghao Lu, Xiaowen Gao, Jun Ou, Jiong Wang

**Affiliations:** ^1^ Department of Spinal Surgery, Affiliated Nanhua Hospital, University of South China, Hengyang, China; ^2^ Health School of Nuclear Industry, Affiliated Nanhua Hospital, University of South China, Hengyang, China; ^3^ Department of Spinal Surgery, Affiliated Hospital of Xiangnan University, Chenzhou, China

**Keywords:** cortical bone trajectory screw, 3D-printing navigation molds, free-hand isthmus method, lumbar internal fixation surgery, retrospective study

## Abstract

**Objectives:**

Traditional cortical bone trajectory (CBT) screws in the lumbar spine offer greater holding strength and are well-suited for patients with osteoporosis. However, the screw implantation procedure is challenging and associated with significant risk. This study aimed to assess whether individualized 3D-printing navigation technology provides higher accuracy and better clinical outcomes compared to the free-hand isthmus method for lumbar CBT screw implantation.

**Methods:**

From September 2020 to August 2023, a total of 41 patients who underwent CBT screw surgery were retrospectively collected. Among them, 23 patients underwent the free-hand isthmus method (Group A), while 18 patients underwent the individualized 3D-printing navigation technique (Group B). All imaging and clinical data for these patients were collected in a blinded manner.

**Results:**

During the surgery, 185 CBT screws were implanted into the lumbar spines of 41 patients—78 in Group A and 107 in Group B. After the surgery, the majority of implanted screws (86.5%) were classified as grade 0, indicating satisfactory implantation. Compared to Group A, Group B had fewer screws classified as grade 1 or grade 2 (*p* = 0.045), indicating higher accuracy in screw implantation. Additionally, Group B also had a shorter operation duration (*p* = 0.02), fewer fluoroscopy exposures (*p* < 0.01), and less blood loss (*p* = 0.03). In addition, compared to Group A, individuals in Group B showed significant improvement in back pain symptoms at both 3 and 6 months (*p* = 0.01 and <0.01), as well as in physical activity at 3 months (*p* = 0.02) postoperatively. No significant difference in postoperative complications was observed between the two groups.

**Conclusion:**

Compared to the free-hand isthmus method, lumbar CBT screw implantation with individualized 3D-printing navigation technology shows higher accuracy, shorter operative time, reduced intraoperative fluoroscopy and blood loss, and better clinical outcomes at three months post-surgery.

## 1 Introduction

The Cortical Bone Trajectory (CBT) is a novel screw insertion technique introduced by Ueno and Santoni in 2009 to address the limitations of traditional pedicle screws (PS), especially in patients with osteoporosis or significant bone loss (1, 2). CBT increases the contact area between the screw and the dense bone cortex, which is largely unaffected by degeneration and osteoporosis, thereby enhancing the screw's holding strength and stability (3–9). Existing evidence shows that CBT offers 30% greater pullout resistance compared to conventional pedicle screws and provides 1.7 times more holding power, even when using shorter screws (10–12).

There are challenges in identifying the correct entry point for CBT. The classical entry point for CBT is located at the intersection of the midline of the superior articular process and 1 mm below the transverse process (1, 2, 12). However, other studies have suggested using the upper edge of the intervertebral foramen as a reference point to reduce the risk of nerve damage (13). Moreover, relying on anatomical landmarks can be challenging in older patients due to joint degeneration, especially when using CBT in individuals with osteoporosis (1–3, 13, 14). Degeneration can make it difficult to identify the entry point accurately, increasing the risk of nerve damage or operator error (15–21). In addition, using the inferior border of the transverse process as a reference increases surgical trauma and becomes particularly challenging when the transverse processes are non-horizontal, especially in patients with a history of previous surgeries. The lumbar isthmus is one of the most easily observable anatomical features during surgical procedures. Due to its proximity to the midline, the isthmus can be fully exposed in spine surgery. Therefore, Paerhati Rexiti et al. suggested a new reference system for entry points based on isthmus parameters (22). However, individual variability in assessing the tangent and distance from the entry point poses a risk of misjudgment in this reference system, which could potentially lead to screw placement errors.

3D-printing technology, an additive manufacturing technique, has been increasingly applied in orthopaedics to improve surgical accuracy (23–25). Recently, researchers have used 3D- printing to create personalized navigation templates for screw implantation, aiming to achieve greater precision (26–28). Shi et al. reported enhanced safety and accuracy when using 3D-printed templates to assist in CBT screw implantation, compared to traditional CBT screws in osteoporotic specimens (29).

However, to the best of our knowledge, there are few studies comparing the clinical outcomes of using individualized 3D-printing navigation technology vs. the free-hand isthmus method in lumbar CBT screw implantation. The aim of this study was to evaluate the intraoperative and postoperative clinical outcomes between these two surgical methods.

## 2 Material and methods

### 2.1 Study population

**Figure 1:**
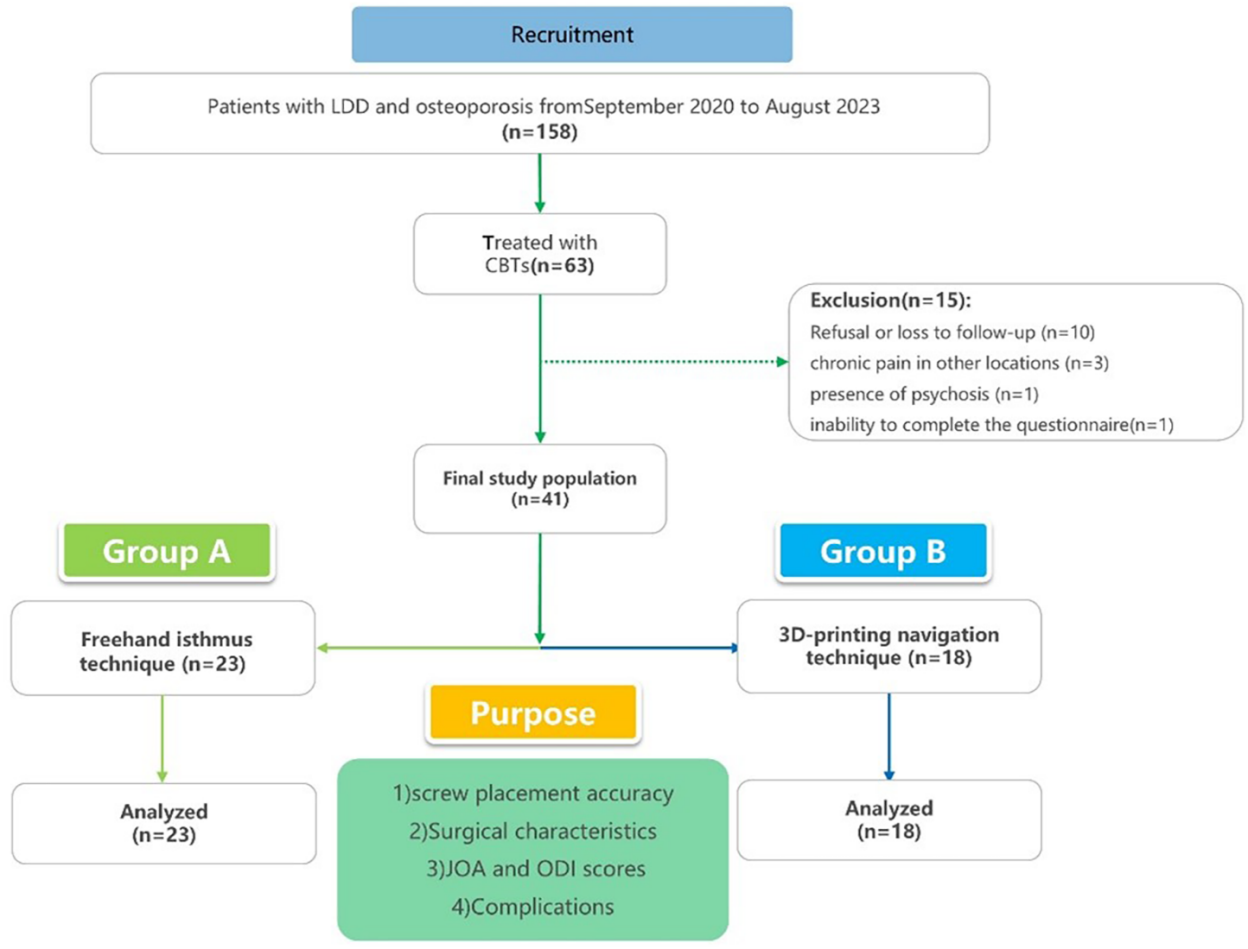
Trials flow diagram.

This single-center retrospective study evaluated the clinical outcomes of patients who underwent surgery for CBT screw implantation between September 2020 and August 2023. The inclusion criteria for this study were as follows: (1) a confirmed diagnosis of lumbar degenerative disease (LDD), osteoporosis (T-score < −2.5), and significant low back pain or neurological deficits unresponsive to conservative treatment; (2) underwent CBT screw implantation surgery using either individualized 3D-printing navigation technology or the free-hand isthums method; and (3) availability of pre- and postoperative data with a minimum follow-up period of 12 months. 56 patients met the inclusion criteria and were initially included in this study. The exclusion criteria included pedicle anomalies, lumbar spondylolysis, secondary osteoporosis, previous lumbar surgeries, and other conditions that prevented surgery or follow-up. 15 patients were excluded due to refusal or loss to follow-up (*n* = 10), chronic pain in other locations (*n* = 3), the presence of psychosis (*n* = 1), or an inability to complete the questionnaire (*n* = 1). In total, 41 patients were included in the final analysis (see Figure 1). The patients were divided into two groups based on the method used for CBT screw implantation: Group A (*n* = 23) utilized the freehand isthmus technique, while Group B (*n* = 18) employed the individualized 3D-printing navigation technique (Figure 1). All surgeries were performed by an experienced senior doctor.

### 2.2 Surgical procedures

In Group A surgeries, the freehand isthmus technique was used to determine the entry point at the intersection of the vertical line extending inward from the isthmus tangent point (ranging from 2.5–5.5 mm, progressively increasing from L1–L5) and the horizontal line of the distal paracentral process. The surrounding bone cortex was ground down, and the screw trajectory was prepared with a head tilt of 25° and an outward tilt of 10°. Mark's localization pin was then placed to fluoroscopically re-confirm the screw trajectory. The trajectory was tapped using a taper 0.5 mm smaller than the planned screw diameter, and a probing pin ensured the integrity of the bone wall within the channel. Once confirmed, screws of the planned length and diameter were inserted along the trajectory, and the procedure proceeded with spinal canal decompression, interbody fusion, and installation of connecting rods as appropriate.

**Figure 2:**
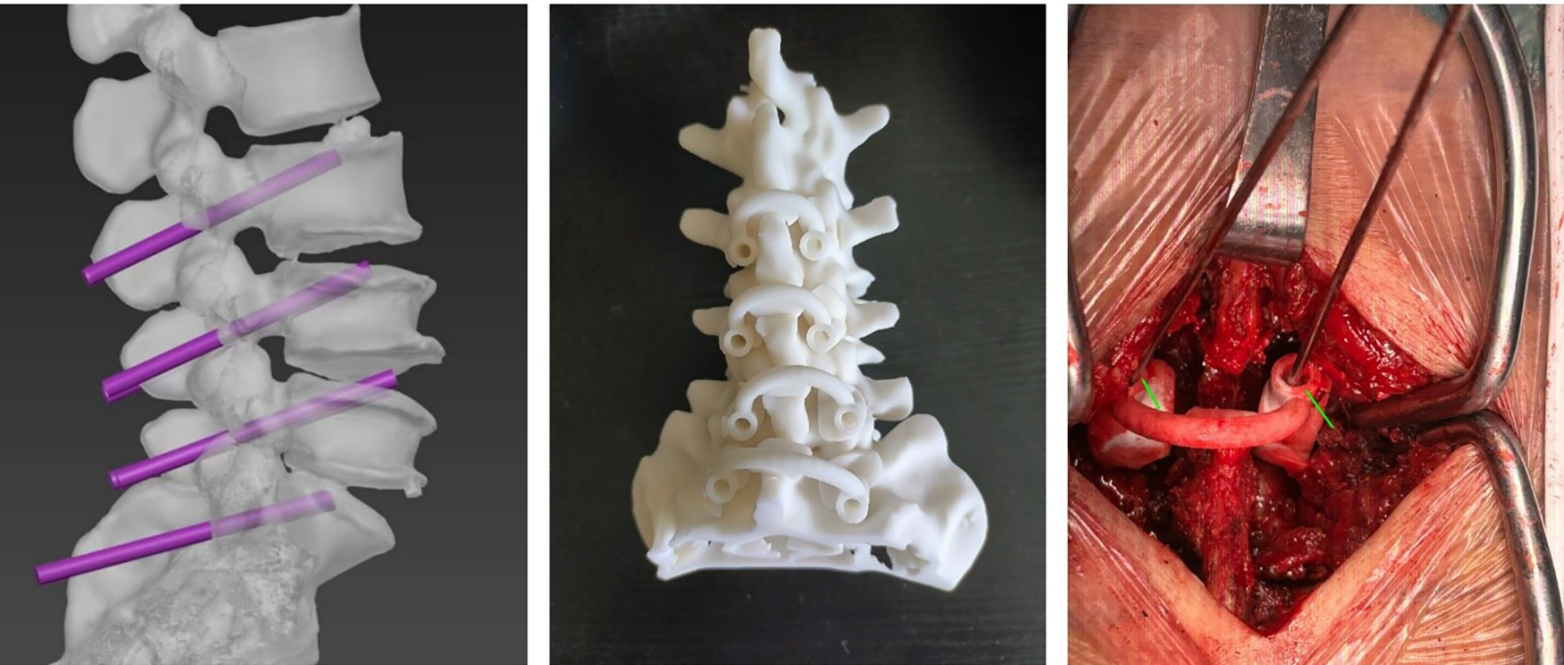
Preoperative and intraoperative steps in lumbar CBT screw implantation using individualized 3D-printing navigation templates. **(A)** 3D-printing navigation templates created using preoperative lumbar CT data; **(B)** CBT screw trajectories planned based on CT data; and **(C)** Intraoperative guidance for lumbar CBT screw implantation using individualized 3D-printing navigation technology.

In Group B surgeries, individualized 3D-printing navigation templates were designed using Mimics Medical software version 21.0 (Materialise Corp., Leuven, VBR, Belgium), Magics software version 24.0 (Materialise Corp., Leuven, VBR, Belgium), and 3D Studio MAX 2020 software(Autodesk Corp., San Francisco, CA, USA), and printed using polyamide fiber. Preoperative lumbar CT data were utilized to create 3D models (Figure 2A), plan cortical bone trajectory (CBT) screw paths, and produce navigation guides (Figure 2B). During surgery, the posterior soft tissues of the vertebral body were carefully stripped to expose the bony surface that aligned with the navigation template. The base of the individualized 3D-printing navigation template was securely fitted onto the cleaned bone surface in the surgical field (Figure 2C). One hand stabilized the template by pressing the curved “handle,” while the other used a power drill to slowly and steadily insert the Kirschner wire through the template guide into the bone at the designated entry point. After drilling to the predetermined depth, the navigation template and Kirschner wire were sequentially removed. The Mark positioning needle was then placed to fluoroscopically confirm the screw trajectory. The subsequent steps, consisting of depth measurement, tapping, checking the integrity of the screw trajectory, inserting the appropriately sized screw, and performing additional surgical procedures (including spinal canal decompression, intervertebral fusion, and installation of connecting rods), were conducted in the same manner as in Group A.

### 2.3 Accuracy of implantation CBT screw

**Figure 3:**
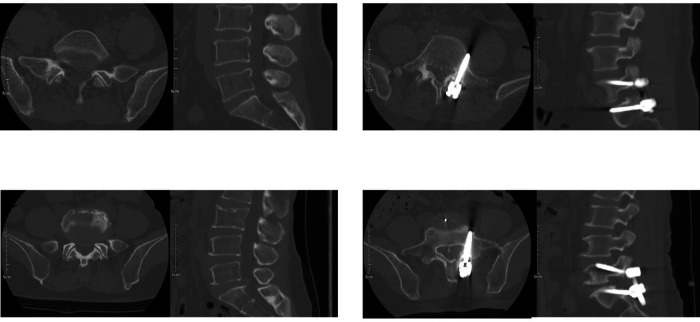
Comparison of preoperative and postoperative CT scans of patients. **(A1)** Preoperative CT of Group A patient: female, 70 years old, lumbar disc herniation; **(A2)** Postoperative CT of Group A patient, through the anterior cortex, Grade 2; **(B1)** Preoperative CT of Group B patient: female, 66 years old, lumbar disc herniation; **(B2)** Postoperative CT of Group B patient, acceptably placed screw, Grade 0.

The primary clinical outcome was the accuracy of screw implantation (Figure 3), which was evaluated by three independent specialists who were not involved in the surgeries. In instances of disagreement, a senior specialist, who was also not involved in the surgeries, acted as the final arbitrator. The CT in our study was a 64-row 128-slice spiral CT system (Siemens Shanghai Medical Equipment Co., Ltd.), and the scanning parameters of the CT machine were uniformly set as follows: tube voltage of 120 KV, tube current of 2 mAS, scanning period of 0.6 s, layer thickness of 0.6 mm, and a matrix of 512 × 512.

Screw implantation accuracy was assessed using the grading system proposed by Ding et al. (30) as follows: Grade 0: The CBT screw is entirely within the pedicle, or the cortical breach on the medial or lateral side is less than half the diameter of the screw. Grade 1: The CBT screw breaches more than half the screw diameter but does not completely penetrate the medial or lateral cortical wall of the pedicle, or there is penetration of the anterior cortex, endplate, or foraminal region. Grade 2: The CBT screw fully breaches the medial or lateral cortical wall. Grade 0 was defined as acceptable screw implantation. The percentage of Grade 0 screws represented the satisfactory screw implantation rate, calculated using the following formula:

Satisfactoryscrewimplantationrate(%)=(NumberofGrade0screws/Totalnumberofscrews)×100%.

### 2.4 Clinical outcome

The secondary clinical outcomes included intraoperative details (the number of fluoroscopy exposures, blood loss, and operation duration), and Japanese Orthopaedic Association (JOA) back symptoms scores, which consist of 14 questions in 4 sections: subjective symptoms, clinical signs, limitations in daily activities, and bladder function. JOA score range: 0–29 (a lower score indicates a greater degree of dysfunction) (31), Oswestry Disability Index (ODI) scores range: 0–100 (a higher scores indicating more severe disability), assessed at 3, 6, and 12 months post-operation, along with monitoring for postoperative complications (32). Common postoperative complications include infection, deep vein thrombosis in the lower limbs, cerebrospinal fluid leaks, and intestinal obstruction.

### 2.5 Statistical analysis

The demographic characteristics of the study population were reported according to the two surgical methods: Individualized 3D-Printing Navigation Technology in Group A and the Free-Hand Isthmus Method in Group B. Categorical variables were presented as case numbers and percentages (%), while continuous variables were presented as mean ± standard deviation (SD). The independent samples *t*-test was used to compare continuous variables between the two groups, while the Chi-square test was utilized for categorical variables. In addition, the Wilcoxon rank-sum test was used to analyze the difference in the satisfactory rate of screw implantation (graded 0–2, %) between the two groups.

Statistical analyses were performed using SPSS software, version 27.0 (IBM Corp., Armonk, NY, USA). A two-tailed *P*-value of less than 0.05 was considered statistically significant.

### 2.6 Ethical approval

The Local Ethical Committee Study approved this study (number: 2020-ky-45). All participants provided written informed consent prior to undergoing surgical treatment.

## 3 Results

A total of 41 patients with an average age of 66.5 ± 6.8 years were included in this study. Of these, 23 (56.1%) underwent free-hand isthmus method (Group A), and 18 (43.9%) underwent individualized 3D-printing navigation technology (Group B) in CBT screw implantation (Table 1). There was no significant difference in age, sex, or BMI between the two patient groups (*p* = 0.81, 0.44 and 0.50, respectively). In addition, the preoperative T-score, JOA score, ODI score, and the number of implant screws used during surgery were also similar between the two groups (*p* = 0.52, 0.96, 0.93 and 0.50, respectively).

**Table 1:** Demographic characteristics of the study population by type of surgical method (*n* = 41).

Demographic characteristics	Surgical method			*P* value
	Entire population	Group A (*n* = 23)	Group B (*n* = 18)	
Age, years	66.5 ± 6.8	66.3 ± 6.6	66.8 ± 7.2	0.81^a^
Sex (male, %)	21 (51.2)	13 (56.5)	8 (44.4)	0.44^b^
BMI (kg/m^2^)	25.8 ± 4.3	26.2 ± 4.4	25.3 ± 4.1	0.50^a^
Pre-operation				
T-score	−3.1 ± 0.3	−3.1 ± 0.3	−3.1 ± 0.4	0.52^a^
JOA score	12.3 ± 1.6	12.3 ± 1.3	12.3 ± 2.0	0.96^a^
ODI score	66.8 ± 6.4	66.9 ± 6.1	66.7 ± 6.9	0.93^a^
During operation				
Number of screws	4.5 ± 1.5	4.7 ± 1.6	4.3 ± 1.4	0.50^a^

Categorical variables were presented as numbers (%), while continuous variables were expressed as mean ± standard deviation.

BMI, body mass index; JOA score, Japanese orthopaedical association score; ODI score, Oswestry disability index score.

^a^ Independent samples *t*-test.

^b^ Chi-square test.

After surgery, the majority of implanted screws (86.5%) were classified as grade 0, indicating satisfactory implantation (Table 2). In Group A, 13 out of 107 screws (12.1%) were classified as grade 1, while 6 out of 107 (5.6%) were classified as grade 2. The satisfactory screw implantation rate in Group A was 82.2% (88 out of 107). In Group B, the proportion of implanted screws classified as grade 1 (5 out of 78, 6.4%) and grade 2 (1 out of 78, 1.3%) was significantly lower (*P* = 0.045). Meanwhile, the satisfactory screw implantation rate in Group B was also significantly higher at 92.3% (72 out of 78) (*P* = 0.048). Additionally, in Group A, the average number of fluoroscopy exposures, blood loss, and surgery duration were 5.3 ± 1.9 times, 241.3 ± 109.4 ml, and 143.1 ± 63.0 min, respectively. In Group B, the number of fluoroscopy exposures (3.1 ± 1.1; *P* < 0.01) and blood loss (172.2 ± 73.2 ml; *P* = 0.03) were significantly reduced. Moreover, surgery duration was also shorter in Group B (101.1 ± 47.0 min; *P* = 0.02).

**Table 2:** Comparison of screw placement accuracy and intraoperative details between the two surgical methods (*n* = 41).

Accuracy of implantation CBT screw	Surgical method			*P* value
	Entire population	Group A (*n* = 23)	Group B (*n* = 18)	
Screw placement accuracy (%)				
Grade 0	160 (86.5)	88 (82.2)	72 (92.3)	0.045^c^
Grade 1	18 (9.7)	13 (12.1)	5 (6.4)	
Grade 2	7 (3.8)	6 (5.6)	1 (1.3)	
Grade 0 screw placement accuracy (%)				
Yes	160 (86.5)	88 (82.2)	72 (92.3)	0.048^b^
No	25 (13.5)	19 (17.8)	6 (7.7)	
Fluoroscopy exposures (times)	4.3 ± 2.0	5.3 ± 1.9	3.1 ± 1.1	<0.01^a^
Blood loss (ml)	211.0 ± 100.3	241.3 ± 109.4	172.2 ± 73.2	0.03^a^
Operation duration (mins)	124.7 ± 59.7	143.1 ± 63.0	101.1 ± 47.0	0.02^a^

Categorical variables were presented as numbers (%), while continuous variables were expressed as mean ± standard deviation.

^a^ Independent samples *t*-test.

^b^ Chi-square test.

^c^ Wilcoxon rank-sum test.

Both groups showed significant improvements in postoperative JOA and ODI scores. In Group A, postoperative JOA scores were 15.3 ± 1.9 at 3 months and 19.5 ± 1.1 at 6 months. In contrast, Group B showed significantly higher postoperative JOA scores at both follow-up time points: 16.9 ± 1.9 at 3 months (*p* = 0.01) and 20.8 ± 1.3 at 6 months (*p* < 0.01) (Table 3). Similarly, the ODI score at the 3-month follow-up was significantly lower in Group B compared to Group A (24.4 ± 6.3 vs. 28.7 ± 5.3, *p* = 0.02). However, no significant difference in long-term postoperative outcomes (e.g., at 12 months) was observed between the two groups. Although no significant difference was found in postoperative complications between the two groups, Group B had a lower complication rate than Group A (*p* = 0.68).

**Table 3:** Post-operative pain intensity, disability, and complications between the two surgical methods (*n* = 41).

Clinical outcome	Surgical method			*P* value
	Entire population	Group A (*n* = 23)	Group B (*n* = 18)	
JOA score after surgery				
The 3rd month	16.0 ± 2.1	15.3 ± 1.9	16.9 ± 1.9	0.01^a^
The 6th month	20.1 ± 1.4	19.5 ± 1.1	20.8 ± 1.3	<0.01^a^
The 12th month	25.0 ± 1.2	25.1 ± 1.2	24.9 ± 1.2	0.62^a^
ODI score after surgery				
The 3rd month	26.9 ± 6.1	28.7 ± 5.3	24.4 ± 6.3	0.02^a^
The 6th month	19.0 ± 4.5	18.6 ± 3.9	19.7 ± 5.1	0.44^a^
The 12th month	15.7 ± 4.0	15.3 ± 4.6	16.1 ± 3.3	0.53^a^
Complications after surgery				
No	35 (85.4)	19 (82.6)	16 (88.9)	0.68^b^
Yes	6 (14.6)	4 (17.4)	2 (11.1)	

Categorical variables were presented as numbers (%), while continuous variables were expressed as mean ± standard deviation.

JOA score, Japanese orthopaedical association score; ODI score, Oswestry disability index score.

^a^ Independent samples *t*-test.

^b^ Chi-square test.

## 4 Discussion

In this retrospective single-center cohort study, we found the following: (1) compared to the free-hand isthmus method, patients with 3D-printing navigation technology had a higher accuracy in screw implantation; (2) patients with 3D-printing navigation technology tended to have fewer fluoroscopy exposures, less blood loss during surgery and shorter operation duration; (3) compared to the free-hand isthmus method, patients with 3D-printing navigation technology reported lower back pain intensity at 3 and 6 months postoperatively, along with improved physical function at 3 months; (4) there was no significant difference in postoperative complications between the two groups.

Santoni (1) proposed the CBT screw technique in 2009, initially for lumbar pedicle implantation. The CBT screw offers several advantages in comparison with conventional techniques, including smaller tissue dissection, greater holding strength, fewer complications, reduced intraoperative bleeding, and lower postoperative infection rates. As a result, it has become a popular focus in spinal surgery research, particularly for patients with osteoporosis and failed pedicle screws fixation. Compared to the classical entry point for CBT, the free-hand isthmus method is symmetrically curved and easily visible during surgery, providing a reliable reference point for screw implantation. Paerhati Rexiti (22) and other researchers suggested using isthmus parameters for screw implantation to reduce tissue damage and the times for x-ray fluoroscopy during spinal surgery. However, variability in the position of the isthmus can lead to increased difficulty of surgery, potential inaccuracies, and an increased risk of failed screw implantation. To the best of our knowledge, this was the first study to explore whether the use of 3D-printing navigation technology, compared to the free-hand isthmus method, in lumbar CBT screw can improve the accuracy of screw implantation and postoperative clinical outcomes.

This study first demonstrated that an effective accuracy of CBT screws implantation (92.3%) in patients with 3D-printing navigation technology, which is relatively higher than that observed in populations with treated with the free-hand isthmus method (82.2%) and some previously reported (1, 33–35). In Group B, six Grade 1 or Grade 2 screws were identified, with four showing lateral cortical perforation and two showing medial perforation, with most perforations occurring within the first half of the study period. In Group A, among the 19 Grade 1 or Grade 2 screws, 14 showed lateral perforations, 4 demonstrated medial perforations and 1 through the anterior cortex. The concept of a “safety zone” between screw perforation and neurovascular complications is well-studied. Generally, a medial cortical perforation margin of up to 4 mm is considered safe (13), while pedicle cortical perforations under 2 mm are deemed acceptable (22, 36, 37), with recommended thresholds of less than 5 mm for medial perforations and less than 6 mm for lateral perforations (22). Anatomically, screws placed medially increase the risk of neurological complications, which leads most surgeons to favor a lateral approach to screw placement. This preference aligns with the higher incidence of lateral cortical perforations observed in this study.

In our study, the accuracy of CBT screw placement using individualized 3D-printing navigation templates exceeded that reported by Federica Penner et al. (38) for single-segment fixation (91.8%), which applied pre-operative CT scan with 3D reconstruction for planning the surgical plan, as well as the results obtained by Kaito et al. (39) and Ke Wang et al. (40) in cadaveric studies using 3D-printing navigation templates (91.4% and 91.6%, respectively). Additionally, compared to Lamartina et al. (41), who reported a 91% accuracy rate for pedicle screws using 3D-printing guides, our results demonstrated a further advantage. However, our accuracy was still lower than that reported by Yue Li et al. (42), where robotic guidance was used to achieve 93% of screws fully contained within the pedicle. However, the use of 3D-printing navigation molds has significant cost advantages over the use of robots for navigation (43).

Our intraoperative observations suggest this discrepancy may stem from inherent instability in the guided manufacturing and application process. Each step—image acquisition, segmentation, 3D modelling, base construction, surface preparation, and guide placement—requires manual intervention, making total standardization challenging. Even minor errors at any stage can create small gaps between the guide and bone surface, reducing stability and affecting screw positioning accuracy.

In our study, except for two patients in Group B, where the guide did not fit the prepared bone surface, requiring a switch to freehand placement, all other navigation templates fit securely. During drilling, the template bases remained firmly fixed, ensuring stable placement. All 185 screws were inserted in both groups successfully without trajectory overlapping.

To further optimize guide stability, surgeons should carefully remove the soft tissue from the bone surface, minimize unnecessary bone damage, and manage osteophytes to ensure a secure fit for the 3D-printing navigation templates.

Meanwhile, patients who underwent surgery with individualized 3D-printing navigation technology showed reduced surgery time, fewer intraoperative fluoroscopy instances, and decreased blood loss. The potential reasons may be attributed to both isthmus parameters and the traditional method of free-hand CBT screw implantation is subjective dependent on the surgeon's experience (44). In contrast, the use of individualized 3D-printing navigation technology allows the surgeon to comprehensively assess the patient's anatomical structure and precisely plan the optimal screw placement path in the preoperative phase (45). 3D-printing guides translate predefined screw placement trajectories directly to the surgical site using solid templates (46). The guide plate features preset apertures that direct the instruments along the correct path with greater accuracy, reducing the potential for errors in the position and angle of screw placement, and mitigating the influence of human factors on the placement trajectory. Moreover, the use of individualized 3D-printing navigation technology reduces the need for intraoperative fluoroscopy to assist with screw positioning, contributing to shorter surgery duration and reduced blood loss (47).

Individuals with lumbar degenerative disease often experienced low back pain and pain related physical disabilities (48). The ODI score, a commonly used measure to assess function in lumbar spine diseases, and the JOA score, a standardized scoring system for human dysfunction, have been widely used to assess lumbar spine function in patients undergoing lumbar fusion and internal lumbar fixation surgery, as an accurate representation of a patient's postoperative recovery (49, 50). In our study, we utilized these scores to assess patient clinical outcomes. The findings of this study indicated that both patient groups experienced significant improvements in pain intensity and pain related physical status, as reflected in their JOA and ODI scores compared to preoperative levels. Specifically, compared with the preoperative status, JOA scores in both groups showed a gradual increase at 3, 6, and 12 months postoperatively, with ODI scores demonstrating a gradual decrease over the same follow-up period, suggesting a favorable surgical outcome, with patients' functional status improving over time, and indicates that postoperative recovery is a progressive process influenced by various factors, which are in line with previous studies (51–53). However, no significant differences were observed between the two groups in ODI and JOA score improvements at all follow-ups except the third month postoperatively (*P* > 0.05), which suggested individualized 3D-printing navigation technology is more beneficial to the short-term outcome of patients but does not affect the long-term outcome of patients significantly. These results may be attributed to several factors: (1) reduced surgical dissection with 3D-printing navigation technology likely minimized surrounding tissues and nerves damage, facilitating better short-term functional recovery and reduced postoperative pain; (2) while precise initial placement was crucial for immediate stability, its impact on long-term recovery may diminish as healing and bone fusion progress similarly in both groups, with long-term changes in JOA and ODI scores influenced by factors including preoperative condition, decompression efficacy, disease duration, and fixation stability, which can offset the early advantages offered by 3D-printing navigation technology. Given the comparable baseline characteristics in both groups, no significant differences were observed at long-term follow-up.

CBT screws were a widely used and effective method for lumbar spine fixation. However, their successful application required a steep learning curve, as surgical outcomes were heavily dependent on the surgeon's expertise. Furthermore, anatomical variations in lumbar vertebrae can affect the accuracy of screw implantation. This study was the first to directly compare the efficacy of 3D-printing navigation technology with the traditional free-hand isthmus technique for CBT screw insertion. Our results suggested that 3D-printing navigation optimizes the surgical process by enabling the preoperative design of the screw path based on patient-specific imaging. However, this approach had some limitations, including the need for advanced planning, additional printing time, and higher costs, making it less suitable for emergency cases.

There were also some limitations in our study. First, the retrospective study design may have led to selection bias and recall bias. Second, the sample size and the fact that all surgeries were performed by the same experienced team from a single institute may limit the generalizability of our findings. Third, the patients included in the analysis had no significant comorbidities and were considered to be at low surgical risk. The study excluded patients with secondary osteoporosis, a history of previous lumbar surgery, infections, or tumors. These factors may affect the external validity of the study results. Finally, this study included only a one-year follow-up for all postoperative patients, limiting its ability to report long-term outcomes and complications. Future research should include a larger and more diverse population and incorporate blinding and well-randomized prospective studies. Once the application of individualized 3D-printing navigation templates for lumbar CBT screws becomes fully established, future studies should focus on further advancements in minimally invasive techniques and expand their use to higher-risk surgical areas, including percutaneous navigation templates and templates for the thoracic and cervical spine. These advancements aim to minimize intraoperative tissue damage, reduce surgical pain and risks, lower surgical costs, and accelerate postoperative recovery.

## 5 Conclusion

Our comparative analysis shows that patients receiving CBT screw implantation with individualized 3D-printing navigation technology achieve higher accuracy, improved operative efficiency, and better clinical outcomes compared to the freehand isthmus method. This new surgical technique could serve as a potential option for patients requiring CBT screw implantation.

## Data Availability

The raw data supporting the conclusions of this article will be made available by the authors, without undue reservation.
